# Artificial intelligence differentiates prefibrotic primary myelofibrosis with thrombocytosis from essential thrombocythemia using digitized bone marrow biopsy images

**DOI:** 10.1038/s41375-026-02893-7

**Published:** 2026-02-20

**Authors:** Andrew Srisuwananukorn, Giuseppe Gaetano Loscocco, James M. Dolezal, Andrew T. Kuykendall, Raffaella Santi, Ling Zhang, Avani M. Singh, Paola Guglielmelli, Alessandro Maria Vannucchi, Mohamed E. Salama, Alexander T. Pearson, Ronald Hoffman

**Affiliations:** 1 Division of Hematology, Department of Internal Medicine, The Ohio State University Comprehensive Cancer Center, Columbus, OH USA; 2 Department of Experimental and Clinical Medicine, ACTIVATE Center, University of Florence, Florence, Italy; 3 CRIMM, Center for Research and Innovation of Myeloproliferative Neoplasms, Azienda Ospedaliero-Universitaria Careggi, Florence, Italy; 4 Geisinger Cancer Institute, Danville, PA USA; 5 Department of Malignant Hematology, H. Lee Moffitt Cancer Center, Tampa, FL USA; 6 Pathology Section, Department of Health Sciences, University of Florence, Florence, Italy; 7 Department of Pathology, H. Lee Moffitt Cancer Center & Research Institute, Tampa, FL USA; 8 Department of Pathology, University of Texas Health Science Center, San Antonio, USA; 9 Sonic Healthcare, Austin, TX USA; 10 Department of Medicine, Section of Hematology/Oncology, The University of Chicago, Chicago, IL USA; 11 Chan Zuckerberg Biohub Chicago, The University of Chicago, Chicago, IL USA; 12 Department of Medicine, Division of Hematology and Medical Oncology, Icahn School of Medicine at Mount Sinai, New York, NY USA

**Keywords:** Myeloproliferative disease, Myeloproliferative disease, Translational research, Diagnosis, Medical imaging

## Abstract

Prefibrotic primary myelofibrosis (prePMF) and essential thrombocythemia (ET) are distinct myeloproliferative neoplasms (MPNs) with overlapping clinical features, often leading to diagnostic uncertainty. We developed an artificial intelligence (AI) framework with human interpretability to distinguish prePMF from ET using digitized hematoxylin and eosin-stained bone marrow biopsy (BMB) slides. Trained on an initial cohort of MPN patients with thrombocytosis, the AI model achieved an AUROC of 0.89 and accuracy of 92.3%. To assess the image features guiding predictions, we generated synthetic images which potentially exaggerate disease-specific morphologies. In a blinded survey, hematopathologists reviewed both real and AI-generated images. While human experts frequently agreed with AI predictions on diagnosis with real images, diagnostic discordance reached up to 88% for AI-generated ET images despite being correctly predicted by AI. We further quantified marrow cellularity and adiposity in the real and generated images, which revealed a higher proportion of fat content in all ET images (42.0%) compared to prePMF (28.9%). These findings suggest that AI can utilize morphological cues distinct from current established diagnostic criteria, such as proportion of adiposity to distinguish types of MPNs. Thus, an AI-assisted diagnostic tool underscores the potential of AI to augment histopathologic evaluation and allow identification of more specific subpopulations of forms of MPNs.

## Introduction

Primary myelofibrosis (PMF) is a clonal stem cell disorder that is recognized as a distinct entity defined by the World Health Organization (WHO) as a Philadelphia chromosome-negative myeloproliferative neoplasm (MPN), which also includes polycythemia vera (PV) and essential thrombocythemia (ET). PMF is further subclassified into “prefibrotic” (prePMF) and overtly fibrotic PMF. Overt MF may also occur during the clinical course of a subset of ET and PV patients, which is termed post-ET and post-PV MF, respectively [1]. The WHO emphasizes the role of megakaryocyte morphology in the classification of the MPNs. Histologic examination of the peripheral blood smear and an adequate bone marrow biopsy (BMB) are indispensable for making the proper diagnosis of PMF. PrePMF was first described as a distinct clinical entity in 1976 by Thiele and colleagues and formally introduced in the 2001 and 2008 WHO diagnostic criteria [2–4]. Then in 2016 and 2022, the WHO provided further guidelines to pathologically distinguish prePMF with thrombocytosis from ET and overt PMF based on megakaryocytic atypia and marrow hypercellularity in the absence of reticulin fibrosis greater than grade 1 [5, 6]. Clinically, prePMF patients may present with isolated thrombocytosis or borderline leukocytosis and is frequently misdiagnosed as ET [7]. Conversely, a subset of ET cases exhibit bone marrow features resembling prePMF. A review of more than 1100 patients initially diagnosed as having ET using the 2008 WHO MPN diagnostic criteria revealed that nearly one in five cases met histopathological criteria for prePMF rather than ET after reassessment of marrow histopathology [8]. Although both disorders are associated with an increased risk of developing thrombotic events, prePMF carries a significantly higher risk and shorter latency period of transformation to overt PMF, leukemia, and death [7–12]. The 2022 WHO and the recent International Consensus Consortium (ICC) MPN diagnostic criteria each define the pathological diagnostic marrow features to differentiate between these two types of MPNs, inherently requiring the subjective assessment of megakaryocyte morphology and fibrosis grading using hematoxylin & eosin (H&E) and reticulin-stained bone marrow biopsies, respectively [13].

As a result of the subjective nature of bone marrow assessments and similar clinical phenotypes, prior reports have identified high rates of discordance among expert pathologists to distinguish ET from prePMF with a modest kappa statistic of 0.474 after participation in a specific MPN education module [14]. At best, diagnostic consensus by groups of pathologists from multiple centers with known expertise in MPN histopathology achieved consensus 88% of the time [15].

The combined recent technical advances within digital pathology and artificial intelligence (AI) offer potential scalable solutions for diagnostic and prognostic assessment across hematological neoplasms [16, 17]. Empiric evidence has shown that AI algorithms may exhibit high degrees of accuracy and reproducibility in image recognition tasks, occasionally surpassing human performance [18]. Much of this progress has been enabled by modern AI architectures including deep convolutional neural networks (DCNNs), transformers, and generative adversarial networks, which have been applied to tasks such as tissue segmentation, cell classification, and morphologic pattern recognition [19, 20]. More recently, foundation models have emerged as promising approach for improving model performance, particularly in data-scarce scenarios. These foundation models are pre-trained on large-scale image datasets, including diverse histopathologic whole slide images, and can be adapted for a wide range of downstream applications. In digital pathology, foundation models previously trained on hundreds of thousands to millions of whole slide images enable improved disease classification, genomic prediction, survival modeling, and other clinically relevant inferences which often achieve near or superhuman performance [21]. Specifically for prePMF and ET differentiation, Ryou et al. developed an AI-derived system termed the “continuous indexing of fibrosis” that closely associated the burden and heterogeneity of fibrosis preferentially with a diagnosis of prePMF. The development of their system focuses on known relevant features such as fibrosis and megakaryocyte morphology [22].

Conversely, there is currently a lack of AI frameworks capable of identifying novel morphological features that would permit accurate distinction of prePMF from ET. For these purposes, publicly available objective-agnostic foundation models pre-trained on large, heterogenous histopathology images offer a promising method for improving diagnostic accuracy. By leveraging knowledge encoded from millions of prior images, foundation models can transfer generalizable representations of histologic patterns to novel or specialized tasks, such as prePMF/ET classification, even when labeled data is scarce. To further understand automatically derived extracted digital biomarkers, our group has shown that the generation of synthetic histopathology can identify human-interpretable morphology not previously characterized for known disease states [23–25].

In this study, we developed an AI model capable of distinguishing prePMF with thrombocytosis from ET on H&E-stained bone marrow biopsy images, with demonstrated potential utility across patient cohorts and institutions. With only knowledge of the marrow images and diagnosis label, our AI model was not specifically trained on known diagnostic features for MPN subtyping.

## Methods

We performed a retrospective, multi-institutional effort to develop and validate an AI algorithm to differentiate prePMF from ET. The custom-written code was developed with the *Slideflow* package written in the Python language, version 3.7 [26]. Computational analyses were performed on High Performance Computing Clusters (HPC) housed within the Icahn School of Medicine at Mount Sinai and the Ohio Supercomputer Center [27].

This research was approved by the Institutional Review Board of Mount Sinai Hospital and The Ohio State University including the study design for algorithmic development of retrospective patient data and pathology images. A waiver for consent was obtained. All experiments were in accordance with the Declaration of Helsinki. The reporting of data is in accordance with AI-specific guidelines including CLAIM and TRIPOD + AI [28, 29].

### Patient Cohorts and Histopathology Digitization

Patients with prePMF and ET were identified at The University of Florence, Italy between 6/2007 and 5/2023, and Moffitt Cancer Center, Florida, USA between 1/2020 and 12/2022. Patients were identified by querying key diagnostic terminology within pathology reports. The diagnosis was revised according to the 2022 WHO and ICC classifications and verified by local expert hemato-pathologists.

De-identified diagnostic H&E-stained BMB core slides were digitized at 40x magnification by Aperio AT2 whole slide scanners (Leica Biosystems). Justification for sample size estimation is available in **Supplemental Methods**.

### Development of complementary AI algorithms for prePMF or ET diagnostic prediction

An AI classification model, hereafter named the prePMF/ET classifier, for automated H&E-stained BMB image analysis to establish a diagnosis of prePMF or ET was developed. The training cohort entailed patients treated at the University of Florence. The model was validated for performance with patients treated at H. Lee Moffitt Cancer Center, Florida, United States.

We developed the prePMF/ET classifier using the open-source software package *Slideflow* with a two-stage approach: feature extraction with a histopathology-informed pre-trained foundation model, followed by attention-based multiple instance learning [26]. The chosen foundation model, RetCCL, was previously trained on 32,000 whole slide images across various cancer subtypes and further underwent re-training upon the described MPN cohorts for diagnosis prediction [30]. We additionally implemented an attention mechanism, which allows the AI model to assign a numeric score to each image region based on how strongly it contributes to the final diagnostic prediction. In effect, attention highlights the areas within the bone marrow biopsy that the model deems most informative for distinguishing prePMF from ET, thereby improving interpretability and enabling visual inspection of the model’s decision-making process [31]. Empirically, attention scores have been shown to improve classification tasks and simultaneously provide a means for qualitative interpretation. For final binary classification, the numeric threshold cutoff to assign a diagnosis was calculated at the point of maximal Youden’s index with the Florence cohort.

**Fig. 1:**
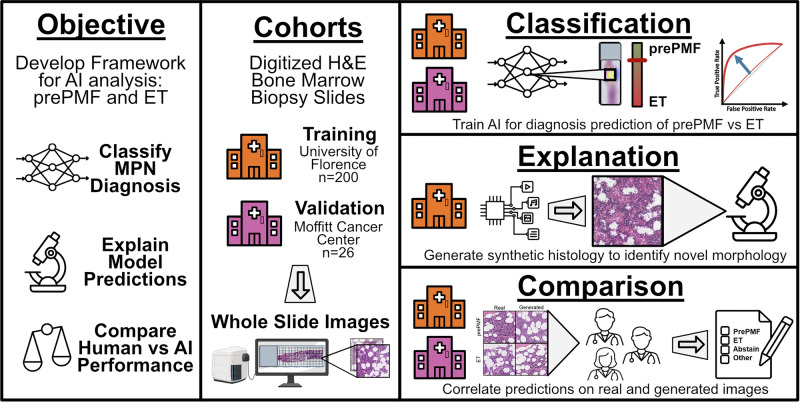
Overview of the current artificial intelligence (AI) strategy for classification and explanation of myeloproliferative neoplasm (MPN) disease prediction. The AI framework was trained on digitized bone marrow biopsies from prefibrotic primary myelofibrosis (prePMF) and essential thrombocythemia (ET) patients treated at the University of Florence, Italy and validated on patients at Moffitt Cancer Center, Florida, United States. A generative AI model was developed to produce synthetic images of prePMF and ET disease states to depict highly relevant morphological features as determined by the classification algorithm. Finally, a survey of real and generated images was evaluated by human hematopathologists and compared with the AI classification model.

To provide additional human interpretation of the prePMF/ET classifier, we developed a second AI system to generate synthetic histology images highly representative of each disease state. We trained a class-conditioned generative adversarial network (cGAN) to exaggerate the subtle morphological features of H&E-stained images of prePMF and ET. We collectively refer to this second explanatory AI system as the prePMF/ET cGAN (Fig. 1). Further technical details regarding the development of both AI systems are provided in **Supplemental Methods**.

### Comparing AI parameters used and hemato-pathologists interpretation of histo-morphologic features

To assess whether the AI-based prePMF/ET classifier performs similarly to human assessment and to understand what relevant parameters AI was using, three hematopathologists (R.S., L.Z., M.E.S.) viewed and assigned a diagnosis for 80 real or generated image tiles that were correctly predicted by the prePMF/ET classifier. During this exercise the allowed diagnostic conclusions included prePMF, ET, abstaining from an answer, or other. The 80 images comprised of real images with highest attention scores extracted from the Moffitt cohort, as well as generated images provided by the prePMF/ET cGAN. The synthetic images were selected with highest attention scores and predicted as the intended diagnosis by the prePMF/ET classifier (i.e., “class-concordant” between classifier and generator). In total, the 80-image survey included 20 images each across real prePMF, generated prePMF, real ET, and generated ET.

The three pathologists were blinded to the source of images (real vs generated) and the true diagnostic labels. Each pathologist’s classification was compared to the prePMF/ET classifier by percent agreement. The proportion of images in agreement was compared using the Fisher’s exact tests.

### Quantifying proportion of adipose tissue and cellularity

We calculated proportion of adipose tissue within each image of the prior 80-image survey by utilizing a custom-trained pixel-level classifier within the open-source *QuPath* software, version 0.5.1 [32]. We trained a model in a similar fashion as Palomaki et al. [33] Within the *QuPath* graphical user interface upon a single real image, we manually annotated regions of adipose tissue or cellularity to train an artificial neural network for pixel-level classification. Training was iterated with additional manually annotated regions until the classification was deemed adequate by visual inspection. The proportion of pixels within each image classified as adipose tissue or cellularity was compared using the Student’s t-test. Of note, areas of artifact, background, or cortical bone were excluded when calculating these proportions. Further hyperparameters for the pixel-level classifier are provided in Supplemental Methods.

## Results

### AI accurately differentiates prePMF from ET using Bone Marrow Biopsy Images

The prePMF/ET classifier was trained on patient specimens from the University of Florence (100 prePMF / 100 ET cases) and externally validated on additional specimens from the Moffitt Cancer Center (6 prePMF / 20 ET cases). Baseline clinical information for the training cohort is provided in Table 1.

**Table 1:** Clinical and laboratory variables at baseline of the University of Florence cohort.

Clinical and laboratory variables at baseline	ET patients (*n* = 100; 50%)	PrePMF patients (*n* = 100; 50%)	*P*-values
Age in year; median (range)	62 (25-92)	64 (18–86)	0.4
Sex, females; *n* (%)	63 (63)	51 (51)	0.09
White blood cells x 10^9^/L; median (range)	8 (4.4–16.5)	9.1 (4.1–36)	**< 0.01**
Hemoglobin, g/dL; median (range)	14.4 (9.9–15.9)	13.7 (9–15.6)	**0.01**
Hematocrit, %; median (range)	43 (35–50)	41.5 (32–50)	**0.01**
Platelets, x10^9^/L; median (range)	624 (452–2000)	640 (220–1660)	0.1
Lactate dehydrogenase, U/L; median (range)	220 (150–403)	283 (157–727)	**< 0.01**
Palpable splenomegaly; *n* (%)	14 (14)	38 (38)	**< 0.01**
**Driver mutations**			
*JAK2*; *n* (%)	76	74	0.6
*CALR* type 1/1 like; *n* (%)	9	13	
*CALR* type 2/2 like; *n* (%)	5	4	
*MPL*; *n* (%)	5	7	
Triple negative*;* *n* (%)	5	2	

*prePMF* Prefibrotic Primary Myelofibrosis, *ET* Essential Thrombocythemia.

**Fig. 2:**
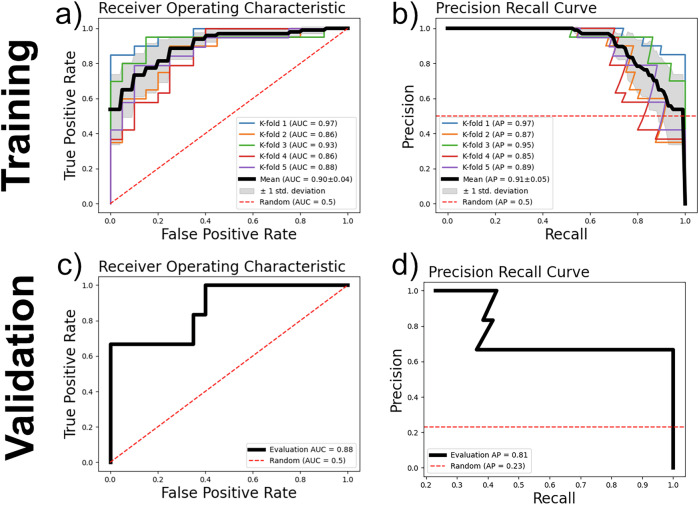
Performance of the prePMF/ET classifier. **a**, **b** Upon the training cohort of patients treated at University of Florence, the model achieved mean area under the receiver operator characteristic (AUROC) of 0.90 ± 0.04 and an average precision (AP) of 0.91 ± 0.05 across 5-fold cross validation. **c, d** Upon external validation of patients treated at Moffitt Cancer Center, the prePMF/ET classifier achieved an AUROC of 0.88 and AP of 0.81. Baseline AP of the external cohort is 0.23, representing the proportion of prePMF diagnoses in the imbalanced cohort.

Using hyperparameters chosen by prior AI histopathology research, the initial models were trained upon 5-fold cross-validation of the Florence cohort to determine preliminary efficacy and consideration of hyperparameter modifications [34]. The classifier models exhibited a mean area under receiver operator curve (AUROC) of 0.90 with a standard deviation of 0.04. The mean average precision (AP) across 5 folds was 0.91 (Fig. 2a, b).

For external cohort assessment, a final classifier model was trained on the full Florence cohort and validated with the Moffitt cohort. After external validation, the classifier achieved AUROC of 0.88 and AP of 0.81, numerically greater than the baseline AP of 0.23 (Fig. 2c, d). To assess the stability of the training process, four additional replicate models were trained with the full Florence cohort and performed similarly with external validation (Supplementary Fig. 2). Finally, we determined the threshold value for binary classification to be the point of maximizing Youden’s Index, which balances sensitivity and specificity when comparing potential threshold values. The resultant fully trained prePMF/ET classifier exhibited a sensitivity of 66.6%, specificity of 100%, and accuracy of 92.3% for prediction of prePMF.

### AI vs Expert Pathologists rely on different histopathological features for assigning the diagnosis of prePMF or ET

**Fig. 3:**
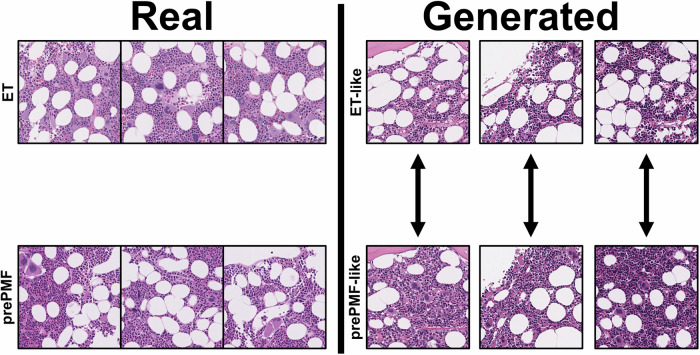
Example image tiles for hematopathologist interpretation survey. Eighty image tiles representing real and generated images from ET or prePMF diagnoses selected for pathologist review. The real image tiles were extracted from the external cohort of patients from Moffitt Cancer Center. The generated images were produced by the prePMF/ET cGAN in tandem, such that a single random seed produced both a highly representative ET-like image or prePMF-like image. Thus, the paired generated images display similar overall architecture but differ in disease-specific morphologies.

With the prePMF/ET classifier model, we aimed to interrogate what morphological features within the images might influence the performance of the model and compare them with human assessments in order to identify if AI and expert pathologists were referencing similar morphological characteristics to render a diagnosis. In this endeavor, we trained a class-conditioned generative adversarial network (cGAN) to generate synthetic images. We previously had shown that generated images can identify new morphological characteristics associated with disease states which may inform human interpretation and thereby improve diagnostic performance [23]. The cGAN was trained upon image tiles extracted at 40x magnification from the training cohort. The fully trained prePMF/ET cGAN generated high-quality synthetic images representing prePMF and ET H&E BMB images (Fig. 3).

To assess the degree of agreement with pathologists and to potentially identify automatically-derived relevant morphological features, a survey of highly representative images— all of which were correctly predicted by the prePMF/ET classifier—were assigned to three expert hematopathologists to determine a diagnosis from a single image tile. The survey of 80 images included real and generated representations of prePMF and ET. Both the prePMF/ET classifier and the hematopathologists predicted a diagnosis without access to information about the clinical context or ability to view additional images from the same “patient”. As such, the expert hematopathologists were allowed to abstain from assigning a diagnosis if it was felt that the provided morphological features in the single image were insufficient to render a diagnosis.

**Fig. 4:**
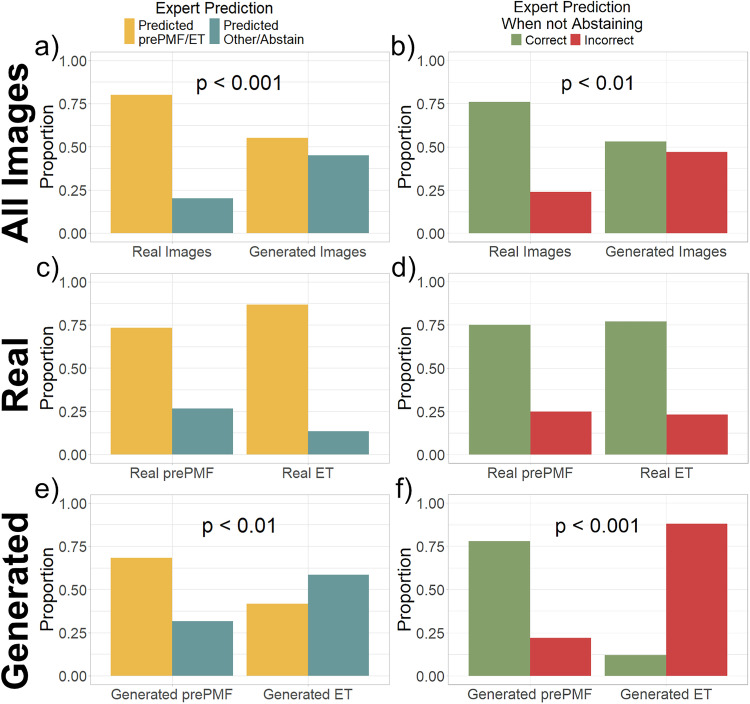
Comparative analysis of hematopathologist assessments of real and generated images. Three hematopathologists were presented with 80 real or synthetic image tiles representing ET or prePMF conditions and were tasked with diagnosing ET or prePMF, abstaining, or proposing an alternative diagnosis. The manually selected images comprised of the subset correctly predicted by the AI classifier. **a**, **b** Across the full set of surveyed images, hematopathologists more often diagnosed real images as ET or prePMF compared to generated ones (80.0% for real vs 55.0% for generated, *p* < 0.001). Among those images where pathologists rendered a diagnosis, accuracy was higher upon the real images (76.0% vs 53.0%, *p* < 0.01). **c**, **d** Within the subset of real images, the assignment of a diagnosis was similar between prePMF and ET (73.3% for prePMF versus 86.7% for ET, *p* = 0.1). When hematopathologists assigned a diagnosis upon real images, the accuracy did not significantly differ with the condition present (75.0% for prePMF vs 76.9% for ET, *p* = 1). **e**, **f** Within the subset of generated image tiles, a higher proportion of prePMF images received a diagnosis by hematopathologists (68.3% vs. 41.7%, *p* < 0.01). Moreover, in the generated images subset with an assigned diagnosis, accurate predictions were substantially more frequent for prePMF images (78.0% vs. 12.0%, *p* < 0.001).

The overall agreement between each of the three hematopathologists with the prePMF/ET classifier was 45.0%; greater agreement was observed with real images (60.8% vs. 29.1%, *p* < 0.001) (Supplemental Table 3). Hematopathologists abstained or assigned an alternative diagnosis after reviewing 32.5% of images. Assignment of an MPN diagnosis by the hematopathologists was more common with real images (80.0% vs. 55.0%, *p* < 0.001). In the subset of images where hematopathologists did not abstain, hematopathologists agreed with the AI-based prePMF/ET classifier more frequently with real images compared to generated images (76.0% vs. 53.0%, *p* < 0.01) (Fig. 4a, b).

**Fig. 5:**
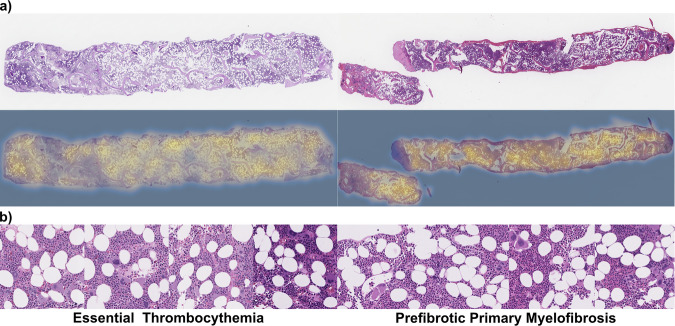
Analysis of high attention areas in bone marrow images. **a** Representative H&E-stained bone marrow biopsy images with associated heatmaps showing attention scores given by the prePMF/ET classifier. In both examples shown, the prePMF/ET classifier accurately identified the underlying diagnosis. The regions highlighted in yellow indicate morphological features that the prePMF/ET classifier deemed significant for the diagnosis. **b** H&E-stained image tiles extracted from the Moffitt cohort exhibiting the top 4 highest attention scores. A manual review confirmed that these areas with high attention scores are associated with areas of hematopoietic cells.

In the subset of real images, the frequency of the hematopathologists abstaining from assigning a diagnosis as well as accuracy of assignment was similar between the real images of prePMF or ET marrow biopsies (Fig. 5c, d). However, in the subset of all generated images, hematopathologists less frequently assigned a diagnosis on generated ET images (MPN diagnosis assigned in 68.3% for prePMF vs 41.7% in ET, p < 0.01). Similarly, the agreement between the hematopathologists and the AI-based prePMF/ET classifier was low for generated ET images (78.0% for prePMF vs. 12.0% for ET images, *p* < 0.001) (Fig. 4e, f). Given that a correct diagnosis was predicted by the prePMF/ET classifier for all samples within the 80-image survey, these findings imply that the generated ET images contain morphological features sufficient for AI but not for expert pathologists to render a diagnosis.

### Human identification of morphology associated with AI prediction

Identification of novel marrow morphologic features associated with the predicted diagnosis was performed at two effective magnification levels. At lower magnification, heatmaps of attention scores produced by the prePMF/ET classifier on the Moffitt cohort allowed for visual assessment of tissue-level image features associated with diagnosis prediction. Interpretation of heatmaps revealed the prePMF/ET classifier’s reliance on areas of hematopoiesis (Fig. 5). Importantly, a qualitative assessment of all heatmaps revealed a lack of high attention to areas of cortical bone, blood clot, or image background/artifactual content across the whole slide images.

**Fig. 6:**
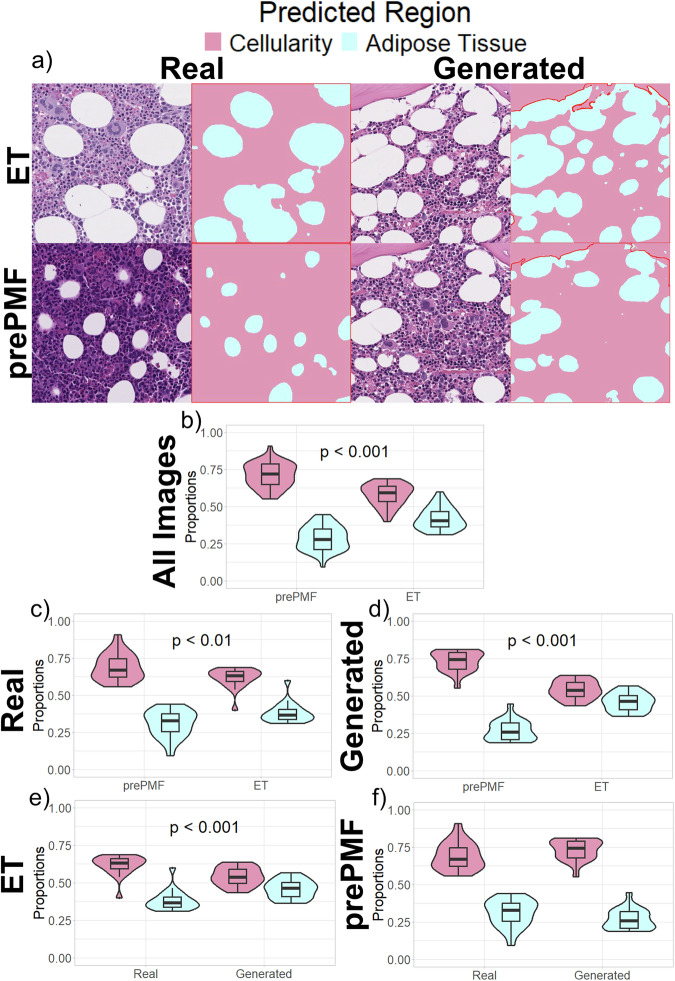
Quantification and comparison of adipose tissue and cellularity on 80 real and generated images. **a** A representative example of H&E-stained images alongside predicted region masks of adipose tissue and bone marrow cellularity. **b–f** When comparing proportions of adipose tissue and cellularity, prePMF images exhibited higher proportions of cellularity and lower proportions of adipose tissue in all images (**b**), the subset of real images (**c**), and the subset of generated images (**d**). Furthermore, there was a lower proportion of cellularity and higher proportion of adipose tissue in the generated ET images compared to real (**e**), and no difference between regions in the subset of real and generated prePMF images (**f**).

At a higher resolution, real and generated image tiles at 40x magnification from the previously described image survey were manually reviewed for cellular morphological features potentially shared between prePMF and ET. Importantly, we did not observe obvious artifactual content such as markings, preparation issues, digitization errors, or other nonbiological image features. Upon initial review, we hypothesized that the proportion of adipose tissue was higher in ET images, and conversely degree of cellularity was higher in prePMF images. Quantification of cellularity and adipose tissue was further performed with a pixel-level classifier trained on manually annotated images (Fig. 6).

In the full survey of 80 real and generated images, the proportion of adipose tissue was determined by the pixel-level classifier to be higher in the ET images (42.0% vs. 28.9%, *p* < 0.001). The trend of higher proportion of adipose tissue in ET was seen in both the subset of real images (38.2% in ET vs. 31.0% in prePMF, *p* < 0.01) and subset of generated images (45.7% vs. 26.8%, *p* < 0.001). As cellularity is inversely correlated to adipose tissue, similar conclusions can be stated that the prePMF images exhibit higher proportions of cellularity (Fig. 6b-d).

To further quantify if degree of cellularity is preserved when generating synthetic images, we compared the proportions of adipose/cellularity within each disease. In the subset of ET images, there was a higher proportion of adipose tissue within generated compared to real images (45.7% vs. 38.2%, *p* < 0.001). However, a significant difference in adipose tissue proportion within the prePMF images was not observed (26.8% in generated vs 31.0% in real, *p* = 0.1) (Fig. 6e, f). These results imply that the generative AI model exaggerates the degree of adipose tissue as a potentially relevant image biomarker for ET but not for prePMF. A representative video of class interpolation between generated ET and prePMF images is provided in Supplemental Video 1.

## Discussion

We present a novel approach using AI to distinguish prePMF from ET based solely on digitized H&E bone marrow biopsy images, which is a challenging diagnostic dilemma for MPN patients with thrombocytosis. Amongst clinicians and hematopathologists, concerns surround the ability of the histomorphologic features to consistently distinguish prePMF from ET, which have led in the past to doubts by some clinicians and pathologists of the actual existence of prePMF as a distinct type of MPN due to the known limited reproducibility of the application of the diagnostic criteria [35]. Due to the close resemblance of the clinical presentations of these two entities, the initial standard of care for patients with these disorders has been routinely chosen by practicing physician to be similar. This practice might be faulty since there are important biological and clinical features which indicate that ET and prePMF are distinct entities and that patients with these disorders might benefit from different treatment approaches.

To address the diagnostic reproducibility issues, several reports have developed models to distinguish ET from prePMF with readily available clinical data. PrePMF patients more frequently present with lower hemoglobin values, higher levels of lactate dehydrogenase, increased white blood cell counts, and palpable splenomegaly. Logistic regression or stepwise decision trees developed from these clinical parameters can identify defined ET with nearly 80% accuracy or 90% positive predictive value with the older 2008 WHO diagnostic criteria [36–38]. New approaches to digital pathology using AI offer additional methods for improved classification of MPN subtypes. Ryou and co-workers first developed an automated AI methodology to objectively quantify fibrosis using routine reticulin-stained BMBs from MPN patients [22]. Their AI algorithm was trained on manually annotated fibrosis images to predict and characterize fibrosis patterns with unseen BMB images. Using an ensemble method of algorithms assessing fibrosis patterns and megakaryocyte morphology, the combined model accurately differentiated prePMF from ET with an internal validation cohort, and furthermore, their combined model was evaluated with ET patients enrolled in the Primary Thrombocythemia-1 (PT-1) clinical trial carried out in the United Kingdom between 1997 and 2012 [39]. The authors determined that the marrow fibrosis distribution identified a subset of ET patients with higher risk for transformation to overt myelofibrosis; given that PT-1 inclusion criteria enrolled patients prior to the formal recognition of prePMF, these findings support the possibility of unintentional enrollment of prePMF patients on to clinical trials, thereby compromising the potential ability to achieve disease-specific endpoints.

By contrast to the AI approach by Ryou and coworkers, our model does not rely on specific preconceived visual features, and as such provides a complementary assessment compared to features delineated by the WHO 2022 and ICC 2022 criteria which emphasize megakaryocyte morphology and the degree of fibrosis. Our model exhibited sustained performance with biopsy specimens procured across academic sites within a multi-institutional collaboration, but future iterations of this algorithm should include real-world biopsies with heterogenous stain quality, preparatory techniques, and clinical manifestations to further interrogate the generalizability of this diagnostic task. We further provided justification of the AI prediction by utilizing a novel generative AI method to identify subtle morphological features associated with each MPN. Generated images of H&E BMBs from these patients also suggested a higher abundance of adipocytes as a variable that could be used to identify ET and conversely cellularity for prePMF. We note that our generative AI model selectively produced images of greater adipose proportion for ET images but not for prePMF. While the identification of novel morphologies by generative AI still requires blinded prospective human evaluation prior to being accepted as a diagnostic standard, it is still noted that the observed difference of adipose content by our generative AI model is in line with earlier investigations noting lower bone marrow fat fraction as determined by T1-weighted magnetic resonance imaging in overt MF patient compared to ET [40]. In addition, the mechanism for decreased adipose tissue in prePMF may mirror the known signaling pathways in overt myelofibrosis cells, where increased levels of the cytokine lipocalin-2 have been shown to diminish adipogenesis of marrow stromal cells ex vivo [41].

In a human vs AI interpretability experiment involving real and generated images, we however noted that expert hematopathologists frequently disagreed with the AI-based model with an overall agreement of 45.0%, and human expert performance upon generated ET images achieved only 12.0% in cases which AI predicted correctly. In spite of this seemingly low performance, it should be noted that expert pathologists were challenged to assign a diagnosis from a single high-magnification image. Similarly, Madelung et al. reported individual agreement for MPN diagnosis with six hematopathologists compared to an expert ground-truth pathologist ranging between 38% and 52% despite having available the full patient biopsy with multiple stains and selected clinical information [14]. These data suggest that full patient bone marrow biopsy images with integrated clinical parameters are necessary for hematopathologists to accurately subclassify MPNs by current diagnostic criteria. Taken together, these findings imply that the AI algorithms and expert pathologists might identify distinctly different morphologies when evaluating H&E bone marrow biopsies. Though we have shown the potential importance of assessing marrow adipocytes in establishing a diagnosis of ET, further work is needed to characterize these subtle visual differences identified by AI.

Overall, the contributions by our group and others developing AI algorithms do not fully represent a substitute for histopathological analysis; these investigations only contribute to increasing the suspicion of a prePMF diagnosis in a patient with a working clinical diagnosis of ET. We envision the potential of an AI tool for automated MPN diagnosis as a valuable aid particularly for providers at centers with lower volumes of MPN patients. Additionally, given its high specificity for prePMF and high sensitivity for ET, future extensions of our proposed AI-based MPN classifier when trained on data from patients with a platelet count > 450 × 10⁹/L may enhance clinical trial enrollment of ET patients by excluding prePMF patients, which would facilitate more accurate assessment of the clinical activity of novel therapeutic agents.

Limitations of the current study include the small sample size of the evaluation cohorts and retrospective nature. Prospective evaluation associating AI prediction with MPN outcomes is warranted. The additional benefit of including other histology stains, such as reticulin, and clinical or molecular data combined with H&E images for AI prediction also warrants additional investigation.

In summary, we have developed a highly accurate AI model that distinguishes prePMF from ET with automated bone marrow analysis. Our model was developed on an open-source framework that could be readily deployed in a rapid and affordable manner for patients with these possible MPN diagnoses.

## Supplementary information

Supplemental Materials

Supplemental Video 1

## Data Availability

The model for classification of prefibrotic primary myelofibrosis and essential thrombocythemia is available at https://github.com/andrewsris/prePMFvsET. For data request inquiries of the whole slide images, please contact the corresponding author (email: Andrew.srisuwananukorn@osumc.edu).

## References

[1] Barosi G, Mesa RA, Thiele J, Cervantes F, Campbell PJ, Verstovsek S, et al. Proposed criteria for the diagnosis of post-polycythemia vera and post-essential thrombocythemia myelofibrosis: a consensus statement from the International Working Group for Myelofibrosis Research and Treatment. Leukemia. 2008;22:437–8. 17728787 10.1038/sj.leu.2404914

[2] Thiele J, Georgii A, Vykoupil KF. Ultrastructure of chronic megakaryocytic-granulocytic myelosis. Blut. 1976;32:433–8. 1064436 10.1007/BF01013883

[3] Vardiman JW, Harris NL, Brunning RD. The World Health Organization (WHO) classification of the myeloid neoplasms. Blood. 2002;100:2292–302. 12239137 10.1182/blood-2002-04-1199

[4] Vardiman JW, Thiele J, Arber DA, Brunning RD, Borowitz MJ, Porwit A, et al. The 2008 revision of the World Health Organization (WHO) classification of myeloid neoplasms and acute leukemia: rationale and important changes. Blood. 2009;114:937–51. 19357394 10.1182/blood-2009-03-209262

[5] Arber DA, Orazi A, Hasserjian R, Thiele J, Borowitz MJ, Le Beau MM, et al. The 2016 revision to the World Health Organization classification of myeloid neoplasms and acute leukemia. Blood. 2016;127:2391–405. 27069254 10.1182/blood-2016-03-643544

[6] Khoury JD, Solary E, Abla O, Akkari Y, Alaggio R, Apperley JF, et al. The 5th edition of the World Health Organization Classification of Haematolymphoid Tumours: Myeloid and Histiocytic/Dendritic Neoplasms. Leukemia. 2022;36:1703–19. 35732831 10.1038/s41375-022-01613-1 PMC9252913

[7] Rumi E, Boveri E, Bellini M, Pietra D, Ferretti VV, Sant’Antonio E, et al. Clinical course and outcome of essential thrombocythemia and prefibrotic myelofibrosis according to the revised WHO 2016 diagnostic criteria. Oncotarget. 2017;8:101735–44. 29254200 10.18632/oncotarget.21594 PMC5731910

[8] Barbui T, Thiele J, Passamonti F, Rumi E, Boveri E, Ruggeri M, et al. Survival and disease progression in essential thrombocythemia are significantly influenced by accurate morphologic diagnosis: an international study. J Clin Oncol. 2011;29:3179–84. 21747083 10.1200/JCO.2010.34.5298

[9] Guglielmelli P, Pacilli A, Rotunno G, Rumi E, Rosti V, Delaini F, et al. Presentation and outcome of patients with 2016 WHO diagnosis of prefibrotic and overt primary myelofibrosis. Blood. 2017;129:3227–36. 28351937 10.1182/blood-2017-01-761999

[10] Gisslinger H, Jeryczynski G, Gisslinger B, Wolfler A, Burgstaller S, Buxhofer-Ausch V, et al. Clinical impact of bone marrow morphology for the diagnosis of essential thrombocythemia: comparison between the BCSH and the WHO criteria. Leukemia. 2016;30:1126–32. 26710883 10.1038/leu.2015.360 PMC4858583

[11] Finazzi G, Carobbio A, Thiele J, Passamonti F, Rumi E, Ruggeri M, et al. Incidence and risk factors for bleeding in 1104 patients with essential thrombocythemia or prefibrotic myelofibrosis diagnosed according to the 2008 WHO criteria. Leukemia. 2012;26:716–9. 21926959 10.1038/leu.2011.258

[12] Passamonti F, Rumi E, Arcaini L, Boveri E, Elena C, Pietra D, et al. Prognostic factors for thrombosis, myelofibrosis, and leukemia in essential thrombocythemia: a study of 605 patients. Haematologica. 2008;93:1645–51. 18790799 10.3324/haematol.13346

[13] Arber DA, Orazi A, Hasserjian RP, Borowitz MJ, Calvo KR, Kvasnicka HM, et al. International Consensus Classification of Myeloid Neoplasms and Acute Leukemias: integrating morphologic, clinical, and genomic data. Blood. 2022;140:1200–28. 35767897 10.1182/blood.2022015850 PMC9479031

[14] Madelung AB, Bondo H, Stamp I, Lovgreen P, Nielsen SL, Falensteen A, et al. WHO classification 2008 of myeloproliferative neoplasms: a workshop learning effect-the Danish experience. APMIS. 2015;123:787–92. 26200697 10.1111/apm.12417

[15] Thiele J, Kvasnicka HM, Mullauer L, Buxhofer-Ausch V, Gisslinger B, Gisslinger H. Essential thrombocythemia versus early primary myelofibrosis: a multicenter study to validate the WHO classification. Blood. 2011;117:5710–8. 21447832 10.1182/blood-2010-07-293761

[16] Srisuwananukorn A, Salama ME, Pearson AT. Deep learning applications in visual data for benign and malignant hematologic conditions: a systematic review and visual glossary. Haematologica. 2023;108:1993–2010. 36700396 10.3324/haematol.2021.280209 PMC10388280

[17] Srisuwananukorn A, Krull JE, Ma Q, Zhang P, Pearson AT, Hoffman R. Applications of artificial intelligence to myeloproliferative neoplasms: a narrative review. Expert Rev Hematol. 2024;17:1–9. 10.1080/17474086.2024.2389997 PMC11996228 39114884

[18] He K, Zhang X, Ren S, Sun J. Delving Deep into Rectifiers: Surpassing Human-Level Performance on ImageNet Classification. 2015 IEEE International Conference on Computer Vision (ICCV) 2015 1026–34 10.1109/iccv.2015.123.

[19] Perez-Lopez R, Ghaffari Laleh N, Mahmood F, Kather JN. A guide to artificial intelligence for cancer researchers. Nat Rev Cancer. 2024;24:427–41. 38755439 10.1038/s41568-024-00694-7

[20] Rashidi HH, Pantanowitz J, Chamanzar A, Fennell B, Wang Y, Gullapalli RR, et al. Generative Artificial Intelligence in Pathology and Medicine: A Deeper Dive. Mod Pathol. 2025;38:100687. 39689760 10.1016/j.modpat.2024.100687

[21] Campanella G, Chen S, Singh M, Verma R, Muehlstedt S, Zeng J, et al. A clinical benchmark of public self-supervised pathology foundation models. Nat Commun. 2025;16:3640. 40240324 10.1038/s41467-025-58796-1 PMC12003829

[22] Ryou H, Sirinukunwattana K, Aberdeen A, Grindstaff G, Stolz BJ, Byrne H, et al. Continuous Indexing of Fibrosis (CIF): improving the assessment and classification of MPN patients. Leukemia. 2023;37:348–58. 36470992 10.1038/s41375-022-01773-0 PMC9898027

[23] Dolezal JM, Wolk R, Hieromnimon HM, Howard FM, Srisuwananukorn A, Karpeyev D, et al. Deep Learning Generates Synthetic Cancer Histology for Explainability and Education, 2022 November 01, 2022:[arXiv:2211.06522 p.]. Available from: https://ui.adsabs.harvard.edu/abs/2022arXiv221106522D. 10.1038/s41698-023-00399-4 PMC10227067 37248379

[24] Hieromnimon HM, Dolezal J, Doytcheva K, Howard FM, Kochanny S, Zhang Z, et al. Building digital histology models of transcriptional tumor programs with generative deep learning for pathology-based precision medicine. Genome Medicine. 2025;4:38. 10.1186/s13073-025-01502-z PMC12329968 40775734

[25] Hieromnimon HM, Trzcinska A, Wen FT, Howard FM, Dolezal JM, Dyer E, et al. Analysis of AI foundation model features decodes the histopathologic landscape of HPV-positive head and neck squamous cell carcinomas. Oral Oncol. 2025;163:107207. 40043423 10.1016/j.oraloncology.2025.107207 PMC13403747

[26] Dolezal JM, Kochanny S, Dyer E, Ramesh S, Srisuwananukorn A, Sacco M, et al. Slideflow: deep learning for digital histopathology with real-time whole-slide visualization. BMC Bioinformatics. 2024;25:134. 38539070 10.1186/s12859-024-05758-x PMC10967068

[27] Center OS. Ohio Supercomputer Center. Ohio Supercomputer Center; 1987.

[28] Mongan J, Moy L, Kahn CE. Checklist for Artificial Intelligence in Medical Imaging (CLAIM): A Guide for Authors and Reviewers. Radiology: Artificial Intelligence. 2020;2:e200029. 33937821 10.1148/ryai.2020200029 PMC8017414

[29] Collins GS, Dhiman P, Andaur Navarro CL, Ma J, Hooft L, Reitsma JB, et al. Protocol for development of a reporting guideline (TRIPOD-AI) and risk of bias tool (PROBAST-AI) for diagnostic and prognostic prediction model studies based on artificial intelligence. BMJ Open. 2021;11:e048008. 34244270 10.1136/bmjopen-2020-048008 PMC8273461

[30] Wang X, Du Y, Yang S, Zhang J, Wang M, Zhang J, et al. RetCCL: Clustering-guided contrastive learning for whole-slide image retrieval. Med Image Anal. 2023;83:102645. 36270093 10.1016/j.media.2022.102645

[31] Ilse M, Tomczak J, Welling M. Attention-based deep multiple instance learning. Proceedings of the 35th International Conference on Machine Learning; 10-15 July 2018; Stockholm, Sweden: PMLR; 2018;80:2127–36.

[32] Bankhead P, Loughrey MB, Fernandez JA, Dombrowski Y, McArt DG, Dunne PD, et al. QuPath: Open source software for digital pathology image analysis. Sci Rep. 2017;7:16878. 29203879 10.1038/s41598-017-17204-5 PMC5715110

[33] Palomaki VA, Koivukangas V, Merilainen S, Lehenkari P, Karttunen TJ. A Straightforward Method for Adipocyte Size and Count Analysis Using Open-source Software QuPath. Adipocyte. 2022;11:99–107. 35094637 10.1080/21623945.2022.2027610 PMC8803053

[34] Dolezal JM, Srisuwananukorn A, Karpeyev D, Ramesh S, Kochanny S, Cody B, et al. Uncertainty-Informed Deep Learning Models Enable High-Confidence Predictions for Digital Histopathology. Nature Communications 2022;13:6572. 10.1038/s41467-022-34025-x PMC9630455 36323656

[35] Barbui T, Thiele J, Vannucchi AM, Tefferi A. Problems and pitfalls regarding WHO-defined diagnosis of early/prefibrotic primary myelofibrosis versus essential thrombocythemia. Leukemia. 2013;27:1953–8. 23467025 10.1038/leu.2013.74

[36] Carobbio A, Finazzi G, Thiele J, Kvasnicka HM, Passamonti F, Rumi E, et al. Blood tests may predict early primary myelofibrosis in patients presenting with essential thrombocythemia. Am J Hematol. 2012;87:203–4. 22237692 10.1002/ajh.22241

[37] Schalling M, Gleiss A, Gisslinger B, Wolfler A, Buxhofer-Ausch V, Jeryczynski G, et al. Essential thrombocythemia vs. pre-fibrotic/early primary myelofibrosis: discrimination by laboratory and clinical data. Blood Cancer J. 2017;7:643. 29233975 10.1038/s41408-017-0006-y PMC5802530

[38] Lekovic D, Bogdanovic A, Sobas M, Arsenovic I, Smiljanic M, Ivanovic J, et al. Easily Applicable Predictive Score for Differential Diagnosis of Prefibrotic Primary Myelofibrosis from Essential Thrombocythemia. Cancers (Basel). 2023;15:4180. 10.3390/cancers15164180 PMC10452817 37627208

[39] Harrison CN, Campbell PJ, Buck G, Wheatley K, East CL, Bareford D, et al. Hydroxyurea compared with anagrelide in high-risk essential thrombocythemia. N Engl J Med. 2005;353:33–45. 16000354 10.1056/NEJMoa043800

[40] Rozman C, Cervantes F, Rozman M, Mercader JM, Montserrat E. Magnetic resonance imaging in myelofibrosis and essential thrombocythaemia: contribution to differential diagnosis. Br J Haematol. 1999;104:574–80. 10086797 10.1046/j.1365-2141.1999.01213.x

[41] Lu M, Xia L, Liu YC, Hochman T, Bizzari L, Aruch D, et al. Lipocalin produced by myelofibrosis cells affects the fate of both hematopoietic and marrow microenvironmental cells. Blood. 2015;126:972–82. 26022238 10.1182/blood-2014-12-618595 PMC4543230

