# Supplementary material for: Artificial intelligence differentiates prefibrotic primary myelofibrosis with thrombocytosis from essential thrombocythemia using digitized bone marrow biopsy images
Source: Leukemia. 2026 Feb 20;40(5):1018–26. doi: 10.1038/s41375-026-02893-7 (PMC13148996; doi:10.1038/s41375-026-02893-7)
Supplement: Supplementary file 1 — Supplemental Materials [file 41375_2026_2893_MOESM1_ESM.docx]

**Supplemental Methods:**

Sample Size Estimation

To determine the sample size needed for training of the prePMF/ET classifier model, we naively assumed a null hypothesis of AUROC of 0.5 and stipulated a power of 80%, one-sided alpha of 0.05, a disease fraction of 0.5 (i.e. 50% of the cohort is prePMF). Using methodology described by Obuchowski,^1^ we would require 31 prePMF and 62 patients total to ensure 80% power to detect an AUROC under the alternative hypothesis of at least 0.7, representing a difference in AUROCs of 0.2.

Image Preprocessing

Each whole slide image (WSI) of a digitized BMB was a gigapixel in size; thus, for computational efficiency, each WSI was tessellated into a library of smaller image tiles prior to model training. Background and artifact were removed by gray space filtering and Gaussian blur detection (sigma of 3 and threshold of 0.02). Given the weakly-supervised nature of the proposed model training framework, automated cell segmentation or region-of-interest annotation was not performed prior to tile extraction.

For training the prePMF/ET classifier model, image tiles were extracted at a width of 302 μm and 299 pixels. Stain normalization was performed using methods described by Macenko et al.^2^ For training the prePMF/ET synthetic image generator model, image tiles were extracted at 300 μm and 512 pixels with no stain normalization performed.

Disease Classifier Training and Validation

The training architecture incorporates two stages as proposed by Niehues et al, which include self-supervised learning and attention-based multiple-instance learning (att-MIL).^3^ In the first stage, image tiles are converted into representative lower-dimensional feature vectors using a histopathology-informed pretrained model. The previously trained model was developed for a WSI retrieval task, named RetCCL, and empirically has been shown to be a generalizable histopathology feature extractor for downstream classification. The RetCCL encoder was trained using self-supervised learning techniques on over 32,000 WSI across multiple tissue types and cancer diagnoses. Using the RetCCL encoder, image tiles are converted into feature vectors with dimensionality of 2048. In the second stage, an att-MIL framework was implemented given its improved performance for automatically detecting sparse relevant morphology.^4,5^ Within an MIL framework, the prediction of a desired outcome occurs in a weakly-supervised fashion upon a collection, or “bag”, of image tile feature vectors. Attention weights are automatically calculated by the model to quantitively assess if certain image tiles are informative in relation to the outcome. Within each bag, the attention scores are scaled by the SoftMax activation function to reduce the range of possible attention values to lie between 0 and 1. Finally, the bag-level feature vector associated with specific slide image is obtained by summing the tile-level feature vectors scaled by each tile’s post-SoftMax attention score. We collectively refer to this system as the prePMF/ET classifier, and hyperparameters for training are provided by **Supplemental Table 1**.

To assess model performance, training occurred by patient-level 5-fold cross validation and also by full cohort training (with a patient-level split of 80%/20%) followed by external validation. To further assess stability, training and validation of the full cohort model was repeated five times. Given stable performance across boot-strapped models, the first repeated model was chosen for further interrogation and explainability.

Initial performance was measured by the area under the receiver operating characteristic (AUROC) and the area under the precision-recall curve (AUPRC). For final binary diagnosis classification, the cutoff threshold value was determined by maximizing Youden’s index (YI) in the validation cohort, which is defined by

$$YI=sensitivity+specificity-1$$

Class conditional Generative Adversarial (cGAN) Network Training

For dataset and model explainability, a cGAN was trained using the StyleGAN2 implementation within the *Slideflow* python package.^6,7^ The cGAN was conditioned by the class label for diagnosis. Class labels were projected into the embedding space and after training, classes were associated with a single numeric embedding vector. The cGAN was not trained with stain normalization or augmentation to allow for improved pathologist interpretation.

For the generation of representative class-conditioned images, a random seed representing noise of 512 x 512 pixels was propagated forward through the fully trained cGAN to produce synthetic prePMF-like or ET-like images.

The cGAN was trained with 4 NVIDIA A100 GPUs upon an HPC compute node for 47,800 kimg (47.8 million total images). Generated images were assessed for subjective image quality prior to training cessation. We collectively refer to this system as the prePMF/ET cGAN, and hyperparameters for cGAN training are provided by **Supplemental Table 2**.

Synthetic Image Tile Generation and Selection

Due to the heterogeneity and sparsity of relevant image features within a training dataset, the unselected generation of synthetic images may not be representative to allow for human interpretation. Thus, random seeds and subsequent generated image tiles were manually chosen where the generated tiles by the prePMF/ET cGAN were deemed to exhibit both high attention and class-concordance with the fully trained prePMF/ET classifier.

First, the cutoff value for “high attention” tiles was determined by calculating the pre-Softmax attention scores across all image tiles within the training cohort (**Supplemental Figure 1**). Upon visual inspection, an attention score of higher than 0.4 was deemed to exhibit “high attention”. Second, high class-concordance was determined if the generated image for a specific diagnosis was also predicted for the specified diagnosis by the fully trained prePMF/ET classifier. If there was diagnostic agreement, the selected image was deemed to exhibit class-concordance between the cGAN and classifier.

Finally, we utilized the interactive user interface *Slideflow Studio* which allows for the simultaneous generation of images by a fully trained cGAN linked with a classification prediction by a fully trained att-MIL classifier. With the *Slideflow Studio* interface, we manually selected highly representative random seeds / generated images for human interpretation. Twenty random seeds were chosen which fulfilled criteria of high attention and class- concordance for both generated prePMF and ET states when interpolating between diagnoses. A representative video of class interpolation between ET and prePMF generated images is provided in **Supplemental Video 1.**

Pixel-level Classifier to Predict Adipose Tissue and Cellularity

Within the *QuPath* user interface, a single real image of ET was manually annotated for regions of adipose tissue and cellularity. A pixel classifier was trained using the Artificial Neural Network Multilayer Perception architecture. The resolution was changed to Full (downsample = 1.00). For improved smoothing, multi-scale features were selected include scaling at 0.5, 1.0, 2.0, 4.0, and 8.0. Other parameters were kept at default levels including red/green/blue channels, Gaussian features, and no local normalization.

**Supplemental Table Legends:**

**Supplemental Table 1:** **Hyperparameters for image preprocessing and training the attention-based multiple instance learning module within the *Slideflow* Python package.** Models were trained by 5-fold cross validation upon the Florence cohort, followed by training upon the full cohort with the same hyperparameters.

**Supplemental Table 2:** **Hyperparameters for image preprocessing and training the class-conditioned generative adversarial network (cGAN).** In total, the cGAN was trained on over 47.8 million total images. Generated images were interpreted at subsequent stages, and training was completed when generated images were deemed to portray high quality. The cGAN was trained on 4 NVIDIA A100 GPUs upon the Ohio Supercomputer, and hyperparameters were chosen to optimize this configuration per NVIDIA labs. Further details can be found at: GPUs <https://github.com/NVlabs/stylegan3/blob/main/docs/configs.md>

**Supplemental Table 3:** **Results for comparative analysis of hematopathologist assessments of real and generated images for prePMF and ET diagnoses.** Three Hematopathologists were tasked to view 20 images each of real or generated images representing prePMF or ET.

**Supplemental Table 1:**

| **Tile Width** | 302 microns |
| --- | --- |
| **Pixel Size** | 299 |
| **Training Backend** | FastAI |
| **Stain Normalizer** | Macenko |
| **Learning Rate** | 0.001 |
| **Weight Decay** | 1e-5 |
| **Bag Size** | 16 |
| **Epochs** | 32 |
| **Batch Size** | 64 |
| **Fit-one-cycle by FastAI** | True |

**Supplemental Table 2:**

| **GAN Backend** | StyleGAN2 |
| --- | --- |
| **Tile Width** | 300 microns |
| **Pixel size** | 512 |
| **Stain Normalizer** | None |
| **Batch Size** | 32 |
| **Batch GPU in Parallel** | 4 |
| **GPU Model** | A100 |
| **Gamma** | 1.6384 |
| **Augmentation Mode** | Ada |
| **Augmentation Pipeline** | bgcfnc (blit, geom, color, filter, noise, cutout) |
| **Mirror** | True |

**Supplemental Table 3:**

|  | **Total Images** | **Predicted Diagnosis Correctly** | **Predicted Diagnosis Incorrectly** | **Abstained** | **Assigned Other Diagnosis** |
| --- | --- | --- | --- | --- | --- |
| Real prePMF | 60 | 33 | 11 | 16 | 0 |
| Real ET | 60 | 40 | 12 | 7 | 1 |
| Generated prePMF | 60 | 32 | 9 | 13 | 6 |
| Generated ET | 60 | 3 | 22 | 30 | 5 |

prePMF: Prefibrotic Primary Myelofibrosis. ET: Essential Thrombocythemia.

**Supplemental Figure Legends:**

**Supplemental Figure 1:** **Histogram of attention scores of all image tiles extracted from the training cohort of patients treated at University of Florence.** Attention scores provided are prior to Softmax activation. Upon visual inspection, a threshold of 0.4 was chosen as a threshold for attention scores. Image tiles with attention greater than 0.4 were deemed to exhibit “high attention.”

**Supplemental Figure 2:** **Repeated training upon the full training cohort of patients treated at the University of Florence with validation upon the external cohort of patients treated at Moffitt Cancer Center**. Performance was stable with mean AUROC 0.89 ± 0.02 and average precision 0.83 ± 0.02 across 5 replicates.

**Supplemental Figures:**

**Supplemental Figure 1:**


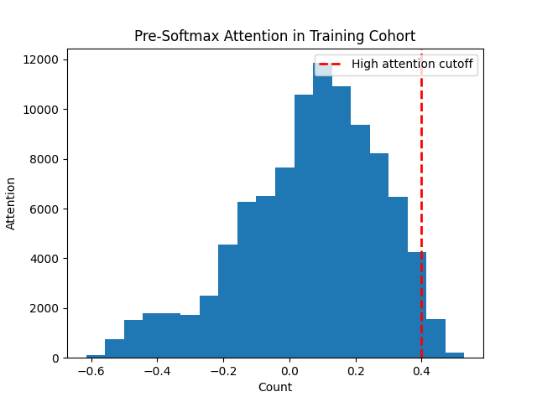


**Supplemental Figure 2:**


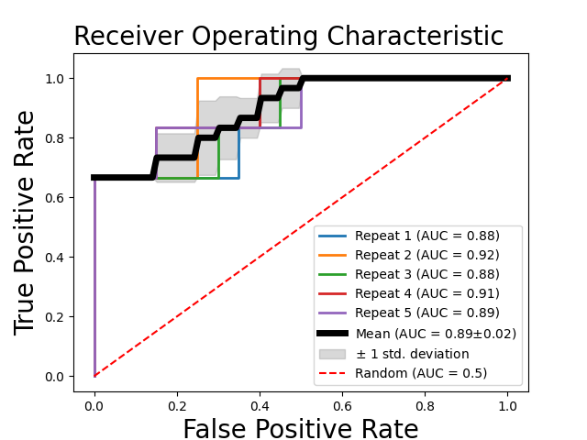

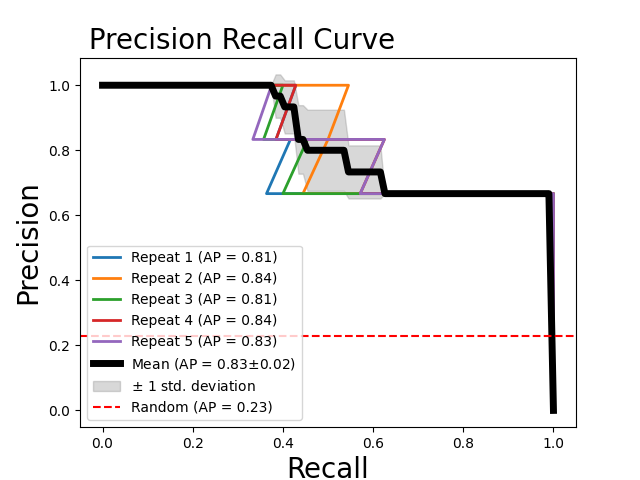


**Supplemental Video Legends:**

**Supplemental Video 1:** **Example video of the *Slideflow Studio* Interface**. In the video, a random seed is chosen to generate an image using the fully trained prePMF/ET cGAN. The model is conditioned by each diagnosis class, producing an image for prePMF-like and ET-like representations. Linear interpolation between disease states allows for the continuous visualization of dynamically changing morphologies, depicted under section “Style Mixing”—Mix=0 represents generated ET-like morphologies and Mix=1 represents generated prePMF-like morphologies. The *Slideflow Studio* interface additionally allows for the simultaneous prediction of a diagnosis from the prePMF/ET classifier, depicted under section “Prediction.” The prePMF/ET classifier displays both the final predicted diagnosis under “bmbx_diagnosis” as well as the automatically assigned attention score for the image. Images with attention greater than 0.4 are deemed to exhibit “high attention.” Utilization of the interface of simultaneous generation and diagnosis prediction allows for users to choose seeds that are both high-attention and class-concordant between the prePMF/ET cGAN and prePMF/ET classifier.

References:

1. Obuchowski NA, McClish DK. Sample size determination for diagnostic accuracy studies involving binormal ROC curve indices. *Stat Med*. 1997;16(13):1529-1542.

2. Macenko M, Niethammer M, Marron JS, et al. A method for normalizing histology slides for quantitative analysis. 2009 IEEE International Symposium on Biomedical Imaging: From Nano to Macro; 2009:1107-1110.

3. Niehues JM, Quirke P, West NP, et al. Generalizable biomarker prediction from cancer pathology slides with self-supervised deep learning: A retrospective multi-centric study. *Cell Reports Medicine*. 2023;4(4):100980.

4. Kather JN, Heij LR, Grabsch HI, et al. Pan-cancer image-based detection of clinically actionable genetic alterations. *Nat Cancer*. 2020;1(8):789-799.

5. Campanella G, Hanna MG, Geneslaw L, et al. Clinical-grade computational pathology using weakly supervised deep learning on whole slide images. *Nat Med*. 2019;25(8):1301-1309.

6. Karras T, Laine S, Aittala M, Hellsten J, Lehtinen J, Aila T. Analyzing and Improving the Image Quality of StyleGAN; 2019:arXiv:1912.04958.

7. Dolezal JM, Kochanny S, Dyer E, et al. Slideflow: deep learning for digital histopathology with real-time whole-slide visualization. *BMC Bioinformatics*. 2024;25(1):134.
